# Associations between CT radiomics analyses and kidney biopsy in patients with kidney disease

**DOI:** 10.1186/s12882-026-05023-8

**Published:** 2026-05-06

**Authors:** Jacob Jalil Hassan, Fabian Baalmann, Jakob Leonhardi, Timm Denecke, Tom H. Lindner, Uwe Scheuermann, Kerstin Amann, Silke Zimmermann, Jonathan de Fallois, Hans-Jonas Meyer

**Affiliations:** 1 Department of Diagnostic and Interventional Radiology, University Hospital Leipzig, 49341/9717400 Leipzig, Germany; 2 Division of Nephrology, Department of Internal Medicine III, University Hospital Leipzig, Leipzig, Germany; 3 Department of Visceral, Transplantation, Vascular and Thoracic Surgery, University Hospital Leipzig, Leipzig, Germany; 4 Department of Nephropathology, University Hospital Erlangen, Friedrich-Alexander-University (FAU) Erlangen-Nürnberg, Erlangen, Germany; 5 Institute of Laboratory Medicine, Clinical Chemistry, and Molecular Diagnostics, University Hospital Leipzig, 04103 Leipzig, Germany

**Keywords:** Kidney failure, Computed tomography, Radiomics, Kidney biopsy

## Abstract

**Background:**

Kidney disease is characterized by microstructural alterations that currently require invasive biopsy for definitive assessment. However, it remains unclear to what extent radiomics features extracted from contrast-enhanced CT can non-invasively reflect kidney function and histopathological changes.

**Methods:**

Between October 2020 and May 2025 all patients undergoing kidney biopsies and having CT scans prior to biopsy were retrospectively analyzed. A total of 49 patients (59% female, median age 60 years) were included. Of the included patients, 35 biopsies were performed in native kidneys (71%) and 14 in kidney allografts (29%). Contrast-enhanced CT images were used to extract radiomics parameters of the kidney. Kidney segmentation was performed using TotalSegmentator and radiomics feature extraction was conducted with PyRadiomics.

**Results:**

Several associations were identified between the extracted radiomics features and kidney function as well as kidney tissue alterations. For the eGFR (CKD-EPI) the highest association was found for the radiomics feature Energy, which is a measurement of the intensity uniformity (ρ = 0.51, p < 0.001), while the first-order feature 90th Percentile showed the best performance in discriminating patients above and below an eGFR threshold of 15 mL/min/1.73 m² with an AUC of 0.83 (95% CI: 0.67-0.98, p = 0.001). Busyness correlated negatively with glomerulosclerosis (ρ = -0.38, p = 0.007), and Coarseness was positively associated with interstitial inflammation (ρ = 0.37, p = 0.008).

**Conclusions:**

CT radiomics features are associated with kidney function as well as histopathological alterations observed in kidney biopsies. Further validation is needed in future studies.

**Supplementary Information:**

The online version contains supplementary material available at 10.1186/s12882-026-05023-8.

## Background

Kidney disease (KD) represents a major global health burden, affecting 10–15% of the adult population worldwide [1]. It is commonly caused by diabetes mellitus, hypertension, and glomerulonephritis, but may also result from hereditary disorders, infections, or drug-induced injury [2]. Regardless of etiology, progressive KD is characterized by a gradual decline in kidney function and can culminate in endstage kidney failure (ESRD). Characteristics of chronic tissue alterations in progressive KD are interstitial fibrosis and tubular atrophy (IFTA), global and focal segmental glomerulosclerosis, and vascular ischemic or arteriosclerotic damage. The extent of these chronic lesions strongly predicts long-term prognosis, independent of laboratory parameters such as estimated glomerular filtration rate (eGFR) or proteinuria [3].

The highest diagnostic quality to assess structural changes in KD is still provided by percutaneous kidney biopsy. However, it is an invasive procedure and carries the risk of complications such as bleeding, macrohematuria, and pain [4].

With the advent of increasingly improved imaging techniques and the rapid development of computer capabilities, the development of non-invasive imaging biomarkers that capture changes in renal microstructure and thus mirror tissue alterations appear feasible. Several non-invasive imaging approaches have been explored, including ultrasound elastography and multiparametric MRI (magnetic resonance imaging) [5, 6]. However, ultrasound is operator-dependent and mainly detects advanced KD, while MRI offers superior sensitivity for fibrosis but is limited by high cost and contraindications [7].

In this context, the emerging field of radiomics harbors significant potential for widespread clinical use. Radiomics refers to the high-throughput conversion of standard medical images into mineable quantitative data by computing a large number of predefined mathematical descriptors, capturing tissue intensity, shape, and texture patterns not discernible to the human eye. These image-derived features can reflect underlying tissue microstructure and pathophysiology. Radiomics techniques have shown particular value in oncology, where image-derived features have been linked to tumor histology and patient outcomes [8]. Beyond oncology, radiomic texture features have also been associated with the degree of fibrosis in organs such as the liver, intestine, and lung [9–11]. Initial studies on computed tomography (CT)-derived radiomics feature extraction in KD provided promising findings [12]. Correlations between radiomics features and histopathological results from kidney biopsies were recently demonstrated [13–14]. The focus of these studies was on non-contrast CT scans. Contrast-enhanced CT, on the other hand, provides more comprehensive information on renal perfusion and tissue heterogeneity, but its radiomics potential in KD remains largely unexplored. Although the intravenous application of contrast agent represents a relative contraindication in patients with impaired kidney function, contrast-enhanced CT is still frequently performed in clinical routine when clinically justified [15].

This exploratory study elucidates whether CT-derived radiomics features can serve as non-invasive surrogates for kidney biopsy findings in KD patients.

A concise overview of the radiomics workflow, key feature categories, and their potential relevance in kidney disease is provided in Supplementary Material S1.

## Materials and methods

### Data collection and study population

The retrospective study was approved by the ethics committee of the University of Leipzig (approval number 249/25ek), and the study was performed in accordance with the relevant guidelines. Informed consent was waived by the ethics committee of the University of Leipzig due to the retrospective nature of the study.

Medical data from adult patients (≥ 18 years of age) who underwent kidney biopsy at the University Hospital of Leipzig between October 2020 and May 2025 were retrospectively screened. Inclusion criteria comprised availability of adequate renal tissue for histopathological assessment and contrast-enhanced CT imaging performed within 6 months prior to biopsy. Patients with incomplete clinical data or insufficient image quality were excluded.

During the study period, 604 patients underwent kidney biopsy. Of these, 49 patients (8.1%) had previously undergone contrast-enhanced CT imaging and were therefore eligible for inclusion in the present study. Among the included patients, 35 biopsies were performed in native kidneys (71%) and 14 biopsies in kidney allografts (29%).

### Image acquisition

Contrast-enhanced CT imaging was performed on a 128-slice or 256-slice scanner (Ingenuity or iCT256, Philips Healthcare, Amsterdam, Netherlands) in the portal-venous phase after 70 s, following intravenous administration of iohexol (Accupaque^®^ 350, GE Healthcare, Chicago, IL, USA). Acquisition parameters included 120 kVp, 36 mAs, collimation of 64 × 0.6 mm, pitch 0.8, and a minimal slice thickness of 1 mm.

### Kidney segmentation

**Fig. 1:**
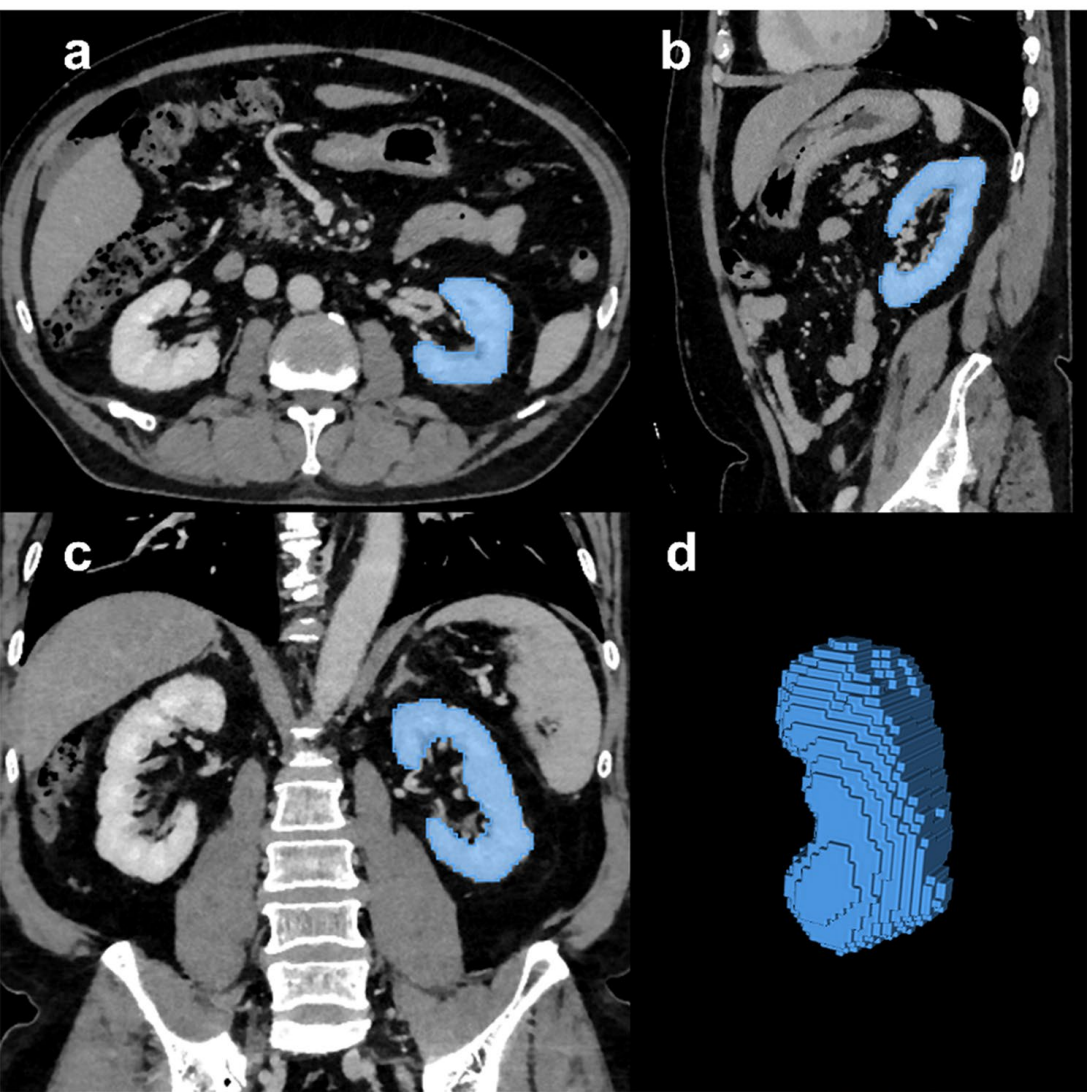
Representative example to demonstrate kidney segmentation. Contrast-enhanced CT of the abdomen. The segmented volume of interest of the left kidney was obtained using the TotalSegmentator plugin in 3D Slicer. (**a**) Axial view, (**b**) sagittal view, (**c**) coronal view, (**d**) three-dimensional voxel volume of the segementation

The anonymized Digital Imaging and Communications in Medicine (DICOM) images were imported into the segmentation software 3D Slicer (version 5.9.0, Brigham and Women’s Hospital, Boston, MA, USA) [16]. Kidney segmentation was performed automatically using the TotalSegmentator plugin, which applies deep learning-based models to robustly segment all major anatomic structures on body CT images [17]. Segmentations were reviewed by a trained reader with 5 years of general experience in radiology, blinded to clinical and histopathological information. Review was performed in 3D Slicer using a standard soft-tissue window (window level 50 HU, window width 400 HU). Only two cases required minor manual boundary refinements, therefore no formal interobserver reproducibility analysis was performed. The kidney that was biopsied was volumetric segmented, in most cases the left side (Fig. 1**).**

### Radiomics feature extraction

Radiomics features were extracted from the kidney segmentations using the PyRadiomics plugin in 3D slicer [18]. All images were resampled to isotropic voxels of 1 mm³ and gray-level discretization was performed with a fixed bin width of 25 Hounsfield Units (HU). Extracted feature classes included first-order statistics, shape3D, shape2D, and higher-order textural features derived from the Gray Level Co-occurrence Matrix (GLCM), Gray Level Run Length Matrix (GLRLM), Gray Level Size Zone Matrix (GLSZM), Gray Level Dependence Matrix (GLDM), and Neighboring Gray Tone Difference Matrix (NGTDM). A total of 111 radiomics features were extracted from every patient.

### Histological analysis and clinical data

Kidney biopsies were part of routine nephrological care to confirm KD with a histopathological diagnosis. All biopsies were performed under ultrasound guidance using an 18-gauge core needle by experienced nephrologists. Standard histological assessment included light microscopy with hematoxylin and eosin- and periodic acid-Schiff-staining, as well as immunohistochemistry and electron microscopy. All biopsies were reviewed and scored by experienced kidney pathologists blinded to the imaging results. Histological findings were graded by quantitative or semi-quantitative scoring. Interstitial inflammation, arteriosclerosis, and acute tubular necrosis (ATN) were scored on a four-point scale: 0 (absent), 1 (mild), 2 (moderate), and 3 (severe). IFTA and global glomerulosclerosis (number of affected/scored glomeruli [%]) were given as percentages. Clinical data and laboratory data closest to the date of biopsy were extracted from patient records. Kidney function was assessed using the estimated glomerular filtration rate (eGFR [mL/min/1.73 m²]), derived from the CKD-EPI formula (Chronic Kidney Disease Epidemiology Collaboration) and quantity of proteinuria (urinary protein-to-creatinine ratio, UPCR [mg/g creatinine]).

### Statistical analysis

Categorical variables were displayed as frequencies with percentages and continuous variables as median with 25th and 75th quantiles or mean with standard deviation (± SD) where appropriate. Multicollinearity among radiomics features was reduced using pairwise Pearson correlation analysis with a cut-off of *r* > 0.8. Radiomics features, clinical parameters, and histopathological scores were evaluated using Spearman’s rank correlation. Receiver operating characteristic (ROC) curve analyses were performed to evaluate diagnostic performance, with area under the curve (AUC) and corresponding 95% confidence intervals (CI) reported. Negative correlations were mathematically inverted prior to model fitting to ensure that higher values indicate a higher risk of histopathological alterations. For eGFR-based ROC analyses, a threshold of 15 mL/min/1.73 m² was used, corresponding to kidney failure according to current international guidelines [19]. Patients, who were on dialysis at the time of biopsy, were excluded from correlation analyses regarding kidney function. For the significant radiomics features of Spearman correlation, a subgroup analysis between native and kidney allografts was performed using Mann-Whitney-U tests. In cases of significant differences between subgroups, the Spearman correlation analysis was subsequently applied again individually. A two-sided p-value < 0.05 was considered statistically significant in all instances. All statistical analyses were performed using SPSS (version 29.0, IBM Corp., Armonk, NY, USA).

## Results

A total of 49 patients were included in this study. Of these, 35 biopsies were performed in native kidneys (71%) and 14 biopsies in kidney allografts (29%). Different underlying causes led to the clinical indication for kidney biopsy, the most common indication was unexplained acute kidney injury (AKI) in 30 cases (61%). At the time of biopsy, nine patients were on dialysis (18%). The median interval between CT imaging and biopsy was 21 days (IQR 7–54). In patients not receiving dialysis, eGFR did not differ significantly between the time of CT (40.8 ± 33.5) and biopsy (41.4 ± 31.4; paired t-test, *p* = 0.99).

Table 1 provides an overview of the patient characteristics.

**Table 1:** Demographic, clinical and histopathological characteristics of the study cohort

Parameter	Distribution
Age, (years)	60 (IQR 41–65)
Sex, n (%)	Male: 20 (41) Female: 29 (59)
Biopsy site, n (%)	Left native kidney: 28 (57) Right native kidney: 7 (14) Kidney allograft: 14 (29)
Indication for kidney biopsy, n (%):	
• Unexplained AKI	30 (61)
• Nephrotic syndrome	4 (8)
• Unexplained CKD	4 (8)
• Decreasing allograft function (not AKI)	2 (4)
• Nephritic syndrome	2 (4)
• Acute kidney disease	2 (4)
• Others	5 (10)
Main histopathological diagnosis, n (%)	
• Interstitial nephritis	8 (16)
• IgA-Nephropathy	6 (12)
• Acute tubular necrosis	5 (10)
• Ischemic	5 (10)
• Thrombotic microangiopathy	4 (8)
• Infection-associated GN	3 (6)
• Pauci-immune crescentic GN	2 (4)
• Membranous nephropathy	2 (4)
• Diabetic nephropathy	2 (4)
• Borderline rejection (allograft)	2 (4)
• Others	10 (20)
Indication for CT imaging, n (%)	
• Tumor staging	17 (34.7)
• Acute abdominal pain	13 (26.5)
• Unclear infection	10 (20.4)
• Kidney transplant follow up	5 (10.2)
• Aortic aneurysm follow up	4 (8.2)
Dialysis-, n (%)	9 (18)
Time interval between CT and biopsy (days)	21 (IQR 7–54)
eGFR at time of CT (mL/min/1.73 m²) *	40.8 (± 33.5)
eGFR at time of biopsy (mL/min/1.73 m²) *	41.4 (± 31.4)
UPCR (mg/g)	983 (IQR 389–5209)
Biopsy results:	
IFTA (%)	10.0 (IQR 5.0-22.5)
Global glomerulosclerosis (%)	4.0 (IQR 0-11.5)
• Interstitial inflammation score 0–3, n (%)	0: 17 (35%) 1: 23 (47%) 2: 6 (12%) 3: 3 (6%)
• Arteriosclerosis score 0–3, n (%)	0: 18 (37%) 1: 10 (20%) 2: 15 (31%) 3: 6 (12%)
• Acute tubular necrosis score 0–3, n (%)	0: 6 (12%) 1: 5 (10%) 2: 15 (31%) 3: 23 (47%)

Values are presented as median (interquartile range [IQR]) or number (percentage). AKI, acute kidney injury; CKD, chronic kidney disease; eGFR, estimated glomerular filtration rate; GN, glomerulonephritis; IFTA, interstitial fibrosis and tubular atrophy; UPCR, urine protein-creatinine ratio. *Excluding patients with dialysis

### Correlation analysis

After testing for multicollinearity radiomics features could be reduced from 111 to 28 singular features. Significant associations of these with laboratory results as well as histopathological alterations were identified.

### Laboratory results

The eGFR correlated positively with the following radiomics features: Energy (ρ = 0.51, *p* < 0.001), 90th Percentile (ρ = 0.50, *p* < 0.001), and 10th Percentile (ρ = 0.50, *p* = 0.001). Cluster Shade (ρ = -0.33, *p* = 0.04) and Low Gray Level Emphasis (ρ = -0.32, *p* = 0.04) were negatively correlated. UPCR showed no significant correlation with any radiomics feature.

### Histopathological parameters

The IFTA-score correlated negatively with 10th Percentile (ρ = -0.34, *p* = 0.02) and Kurtosis (ρ = -0.24, *p* = 0.01). Similarly, global glomerulosclerosis (GS) correlated negatively with Busyness (ρ = -0.38, *p* = 0.007) and Sphericity (ρ = -0.33, *p* = 0.02). For ATN, a negative correlation was observed with Energy (ρ = -0.29, *p* = 0.04) and for arteriosclerosis with Large Dependence Low Gray Level Emphasis (ρ = -0.33, *p* = 0.02) and Minimum (ρ = -0.33, *p* = 0.02).

Interstitial inflammation showed a positive correlation with Coarseness (ρ = 0.37, *p* = 0.008) and negative with Busyness (ρ = -0.33, *p* = 0.02) and Voxel Number (ρ = -0.31, *p* = 0.03). Results of the correlation analyses are summarized in Table 2.

**Table 2:** Spearman correlation analysis of radiomics features with laboratory and histopathological parameters in 49 patients. For analyses involving eGFR and UPCR, dialysis-dependent patients were excluded (*n* = 40). Spearman’s correlation coefficient (ρ) and corresponding p-values are reported

Outcome	Radiomics Feature	Spearman ρ	95% CI	*p*-value
eGFR	Energy	0.51	0.22–0.71	< 0.001*
	90th Percentile	0.50	0.22–0.71	< 0.001*
	10th Percentile	0.50	0.21–0.70	0.001*
	Cluster Shade	-0.33	-0.58-(-0.01)	0.04*
	Low Gray Level Emphasis	-0.32	-0.58-0.00	0.04*
UPCR	Maximum 2D Diameter Column	0.31	0.0-0.57	0.05
	Correlation	-0.30	-0.57-0.02	0.06
IFTA	Kurtosis	-0.24	-0.49-0.05	0.01*
	10th Percentile	-0.34	-0.57-(-0.05)	0.02*
Glomerulosclerosis	Busyness	-0.38	-0.60-(-0.10)	0.007*
	Sphericity	-0.33	-0.57-(-0.05)	0.02*
Acute tubular necrosis	Energy	-0.29	-0.54-0.00	0.04*
	Voxel Number	-0.28	-0.53-0.00	0.05
Arteriosclerosis	Large Dependence Low Gray Level Emphasis	-0.33	-0.57-(-0.05)	0.02*
	Minimum	-0.33	-0.57-(-0.05)	0.02*
Interstitial inflammation	Coarseness	0.37	0.09–0.60	0.008*
	Busyness	-0.33	-0.56-(-0.04)	0.02*
	Voxel Number	-0.31	-0.55-(-0.03)	0.03*

* indicates statistically significant p-values (*p* < 0.05)

### Diagnostic accuracy

ROC analysis was performed to evaluate the discriminatory ability of radiomics features for laboratory and histopathological findings.

For eGFR the first order radiomics feature 90th Percentile had the best discriminatory ability in differentiating patients above and below an eGFR threshold of 15 mL/min/1.73 m² with an AUC of 0.83 (95% CI: 0.67–0.98, *p* = 0.001). Among histopathological parameters, Voxel Number showed the highest diagnostic performance for acute tubular necrosis, discriminating between no/mild versus moderate/severe cases, with an AUC of 0.74 (95% CI: 0.58–0.90, *p* = 0.01). Large Dependence Low Gray Level Emphasis was best at distinguishing patients without arteriosclerosis from those with mild to severe (AUC 0.69, 95% CI: 0.53–0.85, *p* = 0.02). Interstitial inflammation was best predicted by Coarseness, distinguishing mild to severe alterations from no inflammation with an AUC of 0.72 (95% CI: 0.57–0.86, *p* = 0.01).

**Fig. 2:**
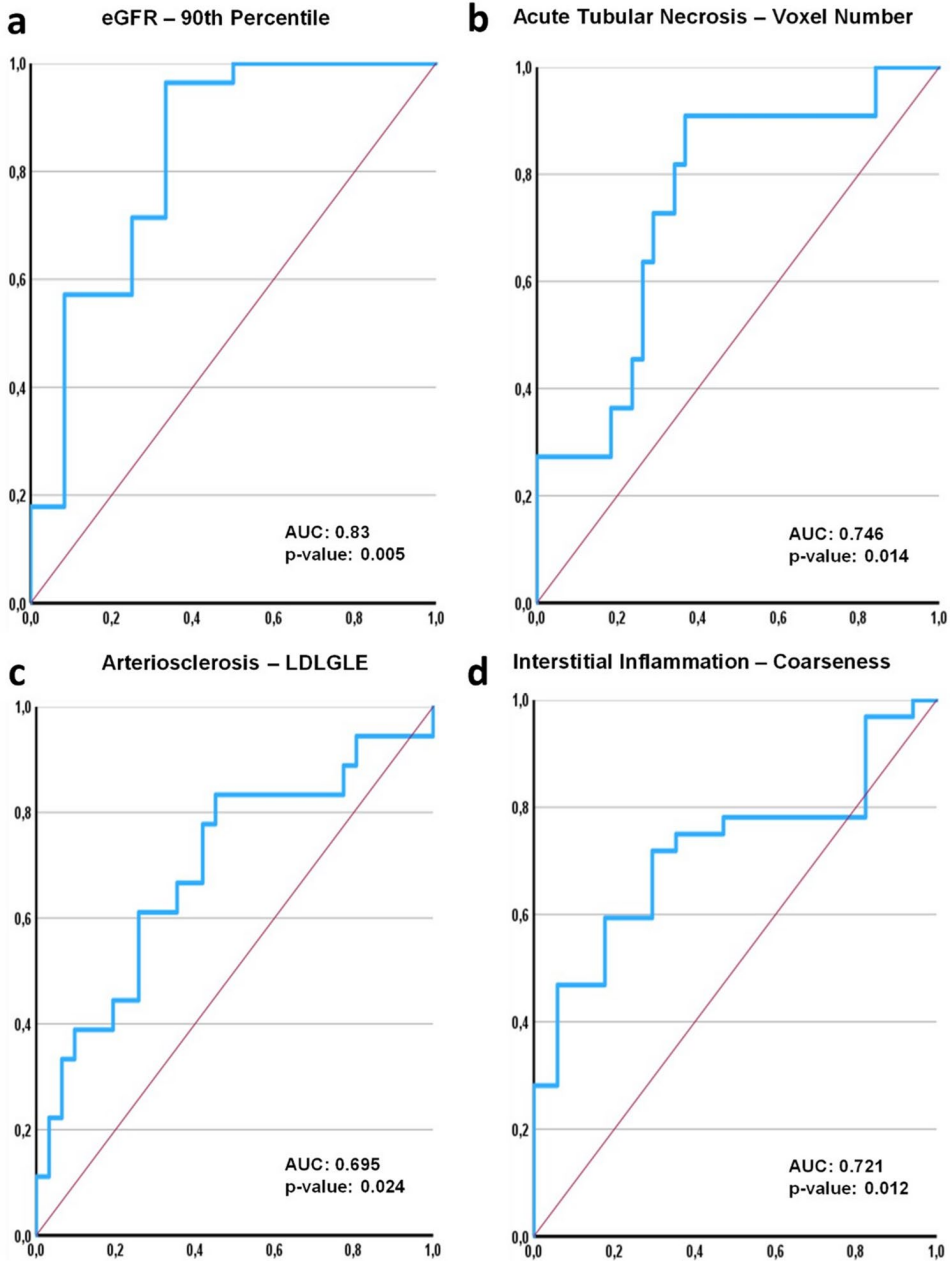
ROC curves for the best-performing radiomics features. ROC curves of the radiomics features with the highest AUC for each laboratory and histopathological parameter. 90th Percentile for eGFR (**a**), Voxel Number for acute tubular necrosis (**b**), Large Dependence Low Gray Level Emphasis (LDLGLE) for arteriosclerosis (**c**), and Coarseness for interstitial inflammation (**d**). Curves were inverted for features with negative correlations in Spearman analysis to maintain consistent interpretation of predictive direction. The x-axis shows specificity and the y-axis shows sensitivity

ROC curve analyses are summarized in Table 3, and the curves for the features with the highest AUC per parameter are illustrated in Fig. 2.

**Table 3:** Receiver operating characteristic (ROC) curve analysis of radiomics features for prediction of laboratory and histopathological parameters in 49 patients

Outcome	Feature	Group comparison	AUC	95% CI	*p*-value
eGFR	10th Percentile	eGFR (cut-off 15 mL/min/1.73 m²)	0.82	0.67–0.97	0.002*
	90th Percentile	eGFR (cut-off 15 mL/min/1.73 m²)	0.83	0.68–0.99	0.001*
	Energy	eGFR (cut-off 15 mL/min/1.73 m²)	0.79	0.64–0.93	0.005*
	Cluster Shade	eGFR (cut-off 15 mL/min/1.73 m²)	0.75	0.60–0.91	0.003*
Acute tubular necrosis	Energy	No/mild/moderate vs. severe	0.68	0.53–0.83	0.03*
	Voxel Number	No/mild vs. moderate /severe	0.75	0.58–0.91	0.01*
Arteriosclerosis	Large Dependence Low Gray Level Emphasis	No vs. mild/moderate /severe	0.70	0.54–0.86	0.02*
	Large Dependence Low Gray Level Emphasis	No/mild vs. moderate /severe	0.68	0.53–0.83	0.03*
Interstitial inflammation	Coarseness	No vs. mild/moderate /severe	0.72	0.58–0.86	0.01*
	Voxel Number	No vs. mild/moderate /severe	0.68	0.53–0.83	0.04*

Dialysis-dependent patients were excluded from analyses involving eGFR and UPCR (*n* = 40). For features with negative correlations in Spearman analysis, curves were inverted to ensure correct interpretation. AUC, area under the curve; CI, confidence interval. * Indicates statistically significant p-values (*p* < 0.05)

### Comparison between native kidneys and kidney allografts

Subgroup analyses between native kidneys and kidney allografts showed significant differences in four radiomics features: Cluster Shade (*p* = 0.01), Voxel Number (*p* = 0.03), Large Dependence Low Gray Level Emphasis (*p* = 0.03), and Coarseness (*p* = 0.03). All other features did not show intergroup differences (Table 4).

**Table 4:** Mann–Whitney U-test results for differences in radiomics features between native kidney (*n* = 35) and transplant kidneys (*n* = 14)

Feature	U	z	*p*	*r*
Cluster Shade	333	2.58	0.01*	0.37
Voxel Number	131	-2.12	0.03*	-0.30
Large Dependence Low Gray Level Emphasis	313	2.12	0.03*	0.30
Coarseness	313	2.12	0.03*	0.30

Values represent test statistics (U, z), significance levels (p), and effect sizes (r). A positive r indicates higher values in the transplant group, a negative r indicates higher values in the native kidney group. * Indicates statistically significant p-values (*p* < 0.05)

Remarkably, individual subgroup Spearman analyses demonstrated significant correlations only for native kidneys: A negative correlation of Voxel Number with acute tubular necrosis (ρ = − 0.37, *p* = 0.02), and of Large Dependence Low Gray Level Emphasis with arteriosclerosis (ρ = − 0.46, *p* = 0.004), as well as a positive one of Coarseness with interstitial inflammation (ρ = 0.50, *p* = 0.002). A negative trend was observed between Cluster Shade and eGFR (ρ = − 0.27, *p* = 0.13), although it did not reach statistical significance. No significant correlations were observed between the radiomics features and outcome parameters in the transplant kidney subgroup (Table 5).

**Table 5:** Spearman correlations between radiomics features and clinical outcomes in native kidneys (*n* = 35) and transplant kidneys (*n* = 14)

Outcome	Feature	Native Kidney ρ (95% CI)	Native Kidney *p*	Transplant ρ (95% CI)	Transplant *p*
eGFR	Cluster Shade	-0.28 (-0.59-0.10)	0.13	-0.30 (-0.81-0.47)	0.43
Acute tubular necrosis	Voxel Number	-0.37 (-0.63-(-0.04))	0.02*	-0.17 (-0.69-0.46)	0.59
Arteriosclerosis	Large Dependence Low Gray Level Emphasis	-0.47 (-0.69-(-0.16))	0.004*	-0.10 (-0.65-0.52)	0.76
Interstitial inflammation	Coarseness	0.50 (0.20–0.72)	0.002*	-0.42 (-0.81-0.22)	0.17

Dialysis-dependent patients were excluded from analyses involving eGFR, resulting in *n* = 31 (native) and *n* = 9 (transplant). Correlations (ρ) and corresponding p-values are reported separately for native kidneys and transplant kidneys. * Indicates statistically significant p-values (*p* < 0.05)

## Discussion

The present exploratory retrospective study demonstrates that quantitative CT radiomics features extracted from contrast-enhanced CT scans are significantly associated with both kidney function and biopsy-proven histopathologic changes.

Irrespective of the underlying etiology, chronic histopathological alterations are key prognostic indicators and can guide therapeutic decision-making [3]. Currently, their assessment is based on invasive kidney biopsy, as noninvasive imaging approaches cannot yet fully exploit their quantitative potential. Fibrosis for example leads to excessive extracellular matrix deposition and disruption of renal microarchitecture [20], which may translate into detectable textural heterogeneity on CT imaging.

The applied methodology is in line with current radiomics standards, using validated and widely available tools. Kidney segmentation was performed with TotalSegmentator, a deep learning–based algorithm that has shown high accuracy across various clinical contexts [21]. Radiomics features were extracted with PyRadiomics, the most established open-source package, ensuring reproducibility according to the Image Biomarker Standardization Initiative (IBSI) [22]. The most informative CT radiomics features in this study were derived from first-order histogram and second-order texture classes, which quantify intrarenal heterogeneity [23].

Notably, several first-order features, like energy, correlated strongly with the current kidney function (eGFR; ρ = 0.51, *p* < 0.001) (Table 2). Energy describes the overall uniformity of voxel intensities within the segmented kidney, with higher values indicating more homogeneous tissue, which may correspond to preserved renal structure. Contrary, the second-order features reflecting textural heterogeneity, were more associated with histopathological alterations: Busyness for example, had inverse correlations with interstitial inflammation and glomerulosclerosis with coefficients of up to -0.38 (*p* = 0.007). Busyness quantifies the rate of local gray-level changes between adjacent voxels, lower busyness thus indicates reduced microstructural variability, potentially reflecting fibrotic remodeling.

Importantly, ROC analyses have shown that individual radiomics features can provide a moderate diagnostic benefit. The 90th Percentile achieved an AUC of 0.83 (*p* = 0.001), which reflects the upper range of renal attenuation values, for differentiating patients with an eGFR below 15 mL/min/1.73 m². Voxel Number, which represents the segmented kidney volume, yielded an AUC of 0.75 (*p* = 0.01) for discriminating no/mild acute tubular necrosis from moderate/severe cases. These findings suggest that radiomics features are associated with underlying microstructural changes in KD. The lack of correlations for UPCR supports this, as it is conceivable that it represents a more dynamic glomerular process that is not adequately captured by static morphological imaging patterns.

When compared to prior radiomics research, our results show good concordance with emerging literature. A recent study from South Korea used non-contrast CT scans of 95 patients and demonstrated significant associations between radiomics features derived from non-contrast CT scans and kidney biopsy findings [14]. The radiomics features predicted glomerulosclerosis, tubular atrophy and interstitial fibrosis with AUCs of 0.65 to 0.74. Similarly, an end-to-end CT radiomics pipeline was recently developed. It predicted interstitial fibrosis grade in KD, achieving an AUC of 0.76–0.804 in a multivariable radiomics model. When clinical variables were added, the combined model outperformed the stand-alone radiomics model, reaching an AUC of 0.918–0.935 [13].

In our present study the AUCs for single radiomics features are in the same range compared to the univariable findings in these studies. The higher AUCs of multivariable analyses reported in these studies reflect the use of more complex models that integrated both radiomics features and clinical parameters, thereby increasing their discriminative power.

Likewise recent MRI-based radiomics studies have also shown strong correlations with kidney function and renal fibrosis. T1 mapping-based radiomics achieved AUCs of up to 0.93 for detecting renal fibrosis, outperforming conventional T1 values [24]. Diffusion derived MRI radiomics have also predicted kidney function and fibrosis with good correlation regarding laboratory results and histopathology [25, 26].

Subgroup analysis between native- and allograft-kidneys confirmed the robustness of the main results. The correlations identified in the total cohort were largely reproducible in native kidneys alone. While a few radiomics features showed significant differences between native and allograft kidneys, the overall correlation patterns remained consistent.

The absence of statistically significant associations in the allograft subgroup is most likely attributable to the limited sample size rather than to biological differences. Hence, it remains to be confirmed in larger cohorts. Preliminary data from CT-, ultrasound-, and MRI-based radiomics analyses indicate that quantitative imaging features may also reflect functional and structural changes in kidney allografts [27–29].

A distinctive aspect of this study is the use of contrast-enhanced CT images acquired during routine clinical practice. This represents a diagnostic advantage, as contrast-enhancement provides additional information that may better capture histopathological alterations which could remain undetectable on non-contrast scans. A direct comparison of radiomics features between non-contrast and contrast-enhanced CT in lung cancer patients demonstrated that a substantial proportion of both first and second order features significantly differ between the two acquisitions, indicating that contrast administration can markedly alter quantitative image metrics [30]. Further merits of the present study are, that it provides insights into real-world clinical data and showed consistent findings across the overall group and subgroups. Furthermore, we found correlations of radiomics features with clinical data that are known to be associated themselves (e.g. eGFR and IFTA/GS), supporting the biological plausibility of our results. Another advantage of using whole kidney radiomics data is that sampling error is significantly reduced since kidney biopsies obtained through a small biopsy needle only capture a minimal portion of the kidney [31]. In addition, the use of standardized, open-source tools for segmentation and feature extraction (TotalSegmentator and PyRadiomics) enhances reproducibility and facilitates future validation in independent cohorts.

Beyond methodological aspects, several additional limitations of the present study should be acknowledged. First, the retrospective design may introduce potential selection, information and reader bias. To mitigate this, kidney segmentation was performed in an automated manner, and the interpreting radiologist was blinded to all clinical information. Second, the time interval between imaging and biopsy varied among patients, reflecting real-world clinical practice but potentially introducing temporal bias. Third, the present analysis included native- and allograft-kidney biopsies. However, subgroup analysis showed largely consistent correlations in native kidneys and similar trends in allografts, indicating that transplant cases did not affect the overall results. Fourth, the intravenous contrast administration is relatively contraindicated in patients with advanced or severe KD [15]. In these patients, administration of iodinated contrast media is associated with an increased risk of contrast-associated acute kidney injury and further decline of residual renal function [32]. Consequently, contrast-enhanced CT in this population requires careful risk-benefit assessment and implementation of preventive strategies, including adequate intravenous hydration, minimization of contrast volume, avoidance of additional nephrotoxic agents, and close post-procedural monitoring of renal function [33]. Fifth, the present study was exploratory in nature. As a result, no correction for multiple comparisons was performed, which may increase the risk of type I error. Furthermore, the lack of reproducibility analyses and external validation limits the generalizability of the observed associations. Accordingly, the findings should be cautiously interpreted as hypothesis-generating. Larger prospective studies with independent external validation cohorts are required to confirm the robustness and clinical applicability of the identified radiomics features.

In conclusion, the results of the present retrospective study suggest that it is possible to use CT-radiomics features to reflect the underlying microstructural changes in KD. This could represent an additional diagnostic benefit for patients in whom kidney biopsy is not possible.

## Supplementary Information

Below is the link to the electronic supplementary material.

Supplementary Material 1

## Data Availability

The datasets generated during the current study are not publicly available but can be obtained from the corresponding author upon reasonable request.

## Abbreviations

AUC: Area under the curve
ATN: Acute tubular necrosis
CKD: Chronic kidney disease
CKD: EPI—Chronic Kidney Disease Epidemiology Collaboration
CI: Confidence interval
CT: Computed tomography
DICOM: Digital Imaging and Communications in Medicine
eGFR: Estimated glomerular filtration rate
GLCM: Gray Level Co—occurrence Matrix
GLDM: Gray Level Dependence Matrix
GLRLM: Gray Level Run Length Matrix
GLSZM: Gray Level Size Zone Matrix
GN: Glomerulonephritis
GS: Global glomerulosclerosis
HU: Hounsfield Unit
IBSI: Image Biomarker Standardization Initiative
IFTA: Interstitial fibrosis and tubular atrophy
IQR: Interquartile range
KD: Kidney disease
KDIGO: Kidney Disease: Improving Global Outcomes
MRI: Magnetic resonance imaging
NGTDM: Neighboring Gray Tone Difference Matrix
ROC: Receiver operating characteristic
UPCR: Urine protein—to—creatinine ratio
