# Supplementary material for: Associations between CT radiomics analyses and kidney biopsy in patients with kidney disease
Source: BMC Nephrol. 2026 May 6;27:295. doi: 10.1186/s12882-026-05023-8 (PMC13154659; doi:10.1186/s12882-026-05023-8)
Supplement: Supplementary file 1 — Supplementary Material 1 [file 12882_2026_5023_MOESM1_ESM.docx]

**Supplementary Material S1.**

**Introduction to Radiomics**

**What is Radiomics?**

Radiomics is a quantitative image analysis approach that converts standard medical images (e.g., CT or MRI) into a large number of mathematically defined features. These features describe image intensity, shape, and texture patterns that are not detectable by visual inspection alone. The underlying premise of radiomics is that imaging phenotypes reflect tissue microstructure and pathophysiology. In contrast to conventional radiological assessment, which is primarily qualitative or semi-quantitative, radiomics enables high-throughput extraction of reproducible imaging biomarkers from routine clinical scans.

**Basic Radiomics Workflow**

A typical radiomics pipeline comprises several consecutive steps. First, standardized CT or MRI images are acquired in routine clinical practice. Subsequently, the region of interest (ROI) is defined through segmentation. Following segmentation, images undergo preprocessing procedures such as resampling to isotropic voxel sizes and gray-level discretization to ensure comparability across patients and scans. Quantitative radiomics features are then extracted using dedicated software tools. Finally, the extracted features are subjected to statistical analysis or modeling and correlated with clinical, laboratory, or histopathological parameters.

**Main Categories of Radiomics Features**

Radiomics features are commonly categorized into first-order, shape, and texture features. First-order (histogram-based) features describe the distribution of voxel intensities within the segmented volume without accounting for spatial relationships. Typical examples include mean and median attenuation values, percentiles (e.g., 10th or 90th percentile), energy as a measure of overall intensity uniformity, and kurtosis or skewness reflecting the shape of the intensity distribution.

Shape features characterize the geometric properties of the segmented organ. These include parameters such as volume (voxel number), surface area, and sphericity.

Texture features, often referred to as second-order or higher-order features, quantify spatial relationships between voxel intensities and thereby capture tissue heterogeneity. They are derived from mathematical matrices such as the Gray Level Co-occurrence Matrix (GLCM), Gray Level Run Length Matrix (GLRLM), Gray Level Size Zone Matrix (GLSZM), Gray Level Dependence Matrix (GLDM), and Neighboring Gray Tone Difference Matrix (NGTDM). Examples include Busyness, describing the rate of local intensity change; Coarseness, reflecting spatial smoothness; Cluster Shade, indicating asymmetry of intensity clusters; and Large Dependence Low Gray Level Emphasis, representing the distribution of low-intensity homogeneous regions.

**Why Radiomics May Be Relevant in Kidney Disease**

Kidney disease is characterized by structural alterations, including interstitial fibrosis, tubular atrophy, glomerulosclerosis, and vascular remodeling. These pathological processes disrupt the normal renal microarchitecture and lead to changes in tissue composition and organization. Although such microstructural alterations are not directly visible on conventional CT imaging, they may influence image intensity distributions and spatial heterogeneity patterns. Radiomics enables the quantitative assessment of these subtle imaging features and may therefore provide non-invasive imaging biomarkers that serve as surrogate markers for biopsy-derived histopathological changes.
